# Etiological relationship between lipid metabolism and endometrial carcinoma

**DOI:** 10.1186/s12944-023-01868-2

**Published:** 2023-08-04

**Authors:** Wenzhe Li, Yi Xu, Xinling Zeng, Jie Tan, Ya Wang, Hongyan Wu, Maokun Li, Cunjian Yi

**Affiliations:** 1 Department of Endocrinology, The First Affiliated Hospital of Yangtze University, Jingzhou, Hubei China; 2 Department of Gynecology and Obstetrics, The First Affiliated Hospital of Yangtze University, Jingzhou, Hubei China; 3 Department of Hematology, The First Affiliated Hospital of Yangtze University, Jingzhou, Hubei China; 4 Department of Hubei Provincial Clinical Research Center for Personalized Diagnosis and Treatment of Cancer, The First Affiliated Hospital of Yangtze University, Jingzhou, Hubei China

**Keywords:** Lipid metabolism, Endometrial carcinoma, Etiological relationship, Statins, Biological mechanisms

## Abstract

Endometrial carcinoma (EC) has become one of the most common gynecological malignant neoplasms in developed countries worldwide. Studies have shown that this may be closely related to the abnormal metabolism of blood lipids, which was the most significant metabolic change in the human body in this cancer. In this review, we focus on the correlation between lipid metabolism and EC and discuss the evidence that abnormal lipid metabolism promotes an increase in EC growth and metabolism, as well as the regulatory mechanism and related signaling pathways involved in this relationship. In addition, we also discussed the research progress of targeted therapies and drug treatments for EC that act on lipid metabolism, and statins are expected to become adjuvant drugs for EC in the future. This review will provide a systematic view for a better understanding of the etiological relationship between lipid metabolism and EC and further open up new therapeutic possibilities and effective treatments for EC by targeting lipid metabolism.

## Introduction

Endometrial carcinoma (EC) has become one of the most common gynecological malignant neoplasms in developed countries worldwide [1, 2]. According to data published by the National Cancer Center in 2022, the incidence rate of EC in China was 10.54/100,000 and the mortality rate was 2.53/100,000. In recent years, the incidence of EC in China has significantly increased due to the increasing standard of living, high-fat, high-sugar, and high-calorie diet, as well as a low-exercise lifestyle [3]. EC is clinically divided into two types according to Bokhman typing, including type I and type II EC [4]. Type I EC is hormone dependent, and the pathological type is mainly endometrioid carcinoma, which has a better prognosis. Type II EC is nonhormone-dependent and mainly includes plasmacytoma, clear cell carcinoma, and carcinosarcoma which has a worse prognosis. Disorders of the reproductive system, infertility, menstrual abnormalities, and genetic factors are significant risk factors for the development of EC. Obesity, abnormal glucose metabolism, and hypertension are also considered to be closely linked to EC and have received a great deal of attention from researchers in the field of tumor metabolism. However, the relationship between lipid metabolism and EC pathogenesis is seldom discussed and is of concern. Therefore, we summarize the relationship between lipid metabolism and EC pathogenesis from the point of view of lipid metabolism.

Lipids are the general term for fats and lipids in blood plasma, including total cholesterol (TC), triglycerides (TG), high-density lipoprotein cholesterol (HDL-C), low-density lipoprotein cholesterol (LDL-C), and other components. Currently, the prevalence of dyslipidemia is reaching a new level of 40.4% among Chinese adults [5]. Dyslipidemia not only increases the risk of cardiovascular disease but also increases the risk of tumor development. Evidence-based medicine research shows that hyperlipidemia can promote the occurrence and development of breast cancer, ovarian cancer, EC, and other malignant tumors [6]. Dyslipidemia is closely related to the development and progression of EC, which is the most significant metabolic change in the human body in this cancer. However, the underlying etiologic mechanisms as well as biological pathways are still unclear. Since the prevention and treatment methods of EC are still not fully effective at this stage, it is of great medical and research value to explore the idea of lipid metabolism mechanisms as a new approach to prevent or delay the development and progression of EC. In this review, we focus on the correlation between lipid metabolism and EC and discuss the evidence that abnormal lipid metabolism promotes an increase in EC growth and metabolism, as well as the regulatory mechanism and related signaling pathways involved in this relationship. Finally, we further discussed the research progress of targeted therapies and drug treatments of EC that act on lipid metabolism to provide information for opening up new therapeutic possibilities and effective treatments for EC by targeting lipid metabolism.

## Epidemiological studies of lipid metabolism and EC

A growing number of epidemiological studies indicate that abnormal lipid metabolism is strongly associated with EC. A cohort study including 13,061 patients noted that dyslipidemia was an independent risk factor for the development of EC, and it significantly increased the incidence of EC. The risk of EC increased by 1.34-fold in patients with elevated TGs (> 150 mg/dL) and 1.65-fold in patients with reduced HDL (HDL < 50 mg/dL) [7]. In addition, the degree of abnormal lipid metabolism increased significantly in EC patients [8]. Another study found that dyslipidemia significantly increased the risk of EC, with a summary OR of 1.622 (95% CI: 1.322–1.989) for hypercholesterolemia (≥ 5.55 mmol/L), 1.250 (95% CI: 1.048–1.490) for hypertriglyceridemia (≥ 1.71 mmol/L), 1.445 (95% CI: 1.164–1.793) for high LDL-C level (≥ 5.50 mmol/L), and 2.401 (95% CI: 1.903–3.029) for low HDL-C level (≤ 1.10 mmol/L) [9]. Furthermore, abnormal lipid metabolism not only increases both the incidence and mortality of EC but also enhances the degree of malignancy of this cancer [10]. The current study found that lower HDL-C levels were associated with an increased mortality risk of EC, with a summary HR of 2.2 (95% CI: 1.1–4.4) [11], whereas elevated TG levels increased the mortality risk of EC by 1.19-fold [12]. Taken together, these data suggest that abnormal lipid metabolism is closely related to the development and progression of EC (Table 1).

**Table 1:** Epidemiological study of lipid metabolism and EC

Incidence or mortality of EC	Lipid Type	No. of cases	Random effects (95%CI)	*P* value	Ref
Incidence	HDL-C (mg/dL) <50	90	1.66 (1.22–2.03)	< 0.050	[7]
	HDL-C (mg/dL) <42	318	2.40 (1.90–3.03)	< 0.001	[9]
	LDL-C (mg/dL) >213	182	1.45 (1.16–1.79)	< 0.001	[9]
	TG (mg/dL) >151	287	1.25 (1.05–1.49)	0.014	[9]
	TC (mg/dL) >215	203	1.62 (1.32–1.99)	< 0.001	[9]
Mortality	HDL-C (mg/dL) <39	153	2.20 (1.10–4.40)	0.034	[11]

EC, endometrial carcinoma; TG, triglycerides; TC, total cholesterol; LDL-C, low-density lipoprotein cholesterol; HDL-C, high-density lipoprotein cholesterol

## Biological mechanisms linking lipid metabolism and EC

In recent years, increasing evidence has shown that dyslipidemia is strongly associated with the development and progression of EC, but the underlying etiologic link between dyslipidemia and EC is less well understood. Although the exact biological mechanisms and pathophysiological events have not been fully investigated, the potential mechanisms, including different types of dyslipidemia, obesity, inflammation, angiogenesis, estrogen metabolism, and other specific activation of signaling pathways involved, may give rise to an excess risk of EC in patients with abnormal lipid metabolism.

### Lipids and ECs

#### TG and EC

An important component of serum lipids is triglyceride, which is the main component of adipose tissue, and high levels of TG will accumulate in adipocytes. The accumulated fat then produces large amounts of aromatase, which converts androstenedione to estradiol, resulting in elevated estrogen levels [8, 13]. On the other hand, high levels of TG also inhibit the production of sex hormone-binding globulin (SHBG), which reduces the binding state of estrogen and contributes to a further elevation of functioning estrogen levels. Elevated levels of estrogen not only mediate EC cell proliferation and angiogenesis but also inhibit their apoptosis, which finally leads to EC progression [14]. It has been reported that the expression of peroxisome proliferator-activated receptor γ (PPARγ) is downregulated in EC as well as several other malignancies, suggesting that PPARy may be an oncogene [15]. It is worth noting that high levels of TG may cause a decrease in PPARγ levels, thus leading to the occurrence of EC [16]. In addition, another key subtype of peroxisome proliferator-activated receptor (PPAR), PPARα, also plays an important role in EC genesis. In a previous animal experiment, researchers established a mouse model fed PPARα ligands and found increased levels of CDK4 and CDK1 proteins in the liver and liver carcinogenesis in mice [17]. Elevated levels of PPARα were also found in EC; since TG acts as a ligand of PPARα, it is suggested that one possible risk link between TG and EC could be through influencing the levels of PPARα [15, 18].

Excess TG in the body will accumulate abnormally in the liver and be accompanied by an increase in free fatty acids in the liver, thus forming a fatty liver, leading to damage to liver function as well as insulin resistance (IR) [19]. In the state of IR, the effect of insulin on the inhibition of fat degradation is diminished, and free fatty acids are further activated by serine kinase, thus aggravating the degree of IR and leading to hyperinsulinemia [20]. In vivo, hyperinsulinemia may induce the action of EC in multiple malignant phenotypes through the activation of several important receptor-mediated pathways, such as insulin receptors and insulin-like growth factor 1 receptor (IGF-1R), and the typical signaling pathways involved in these receptors include the phosphatidylinositol 3-kinase (PI3K)/extracellular signal-regulated kinase (ERK/AKT) and mitogen-activated protein kinase (MAPK) pathways [21, 22]. The apolipoprotein E (AopE) family is closely related to EC development, and it has been indicated that AopE triggers the activation of the ERK/matrix metalloproteinase 9 (MMP9) signaling pathway, which subsequently facilitates the migration of EC cells. Additionally, the increased MMP9 protein compromises the integrity of the basement membrane, thereby facilitating tumor invasion and metastasis. However, the knockout of the ApoE gene significantly suppresses these processes [23, 24]. It is worth noting that patients with hypertriglyceridemia tend to exhibit AopE2 in their plasma. Nevertheless, the relationship between apolipoprotein subtypes and EC is unclear. Therefore, further research is needed to uncover the underlying mechanism by which TG influences EC through Apoe2.

#### TC and EC

Serum cholesterol levels ≥ 200 mg/dL were found to increase the risk of endometrial lesions by 1.8-fold [25]. One of the most closely related to the pathogenesis of EC is 27-hydroxycholesterol (27HC), which is one of the metabolites of TC. It is mainly synthesized by the CYP27A1 enzyme and catabolized by the CYP7B1 enzyme. Studies have indicated that 27HC can act on estrogen receptors and affect the growth and metastasis of hormone-sensitive ER-positive breast cancer cells by activating the liver X receptor (LXR) [26–28]. It has been reported that LXR is widely present in tissues and has been proposed as a new anticancer target, supported by the results of numerous breast cancer studies. It is worth noting that the expression of the CYP7B1 enzyme was significantly decreased, while CYP27A1 and 27HC levels were elevated in the low differentiated EC group compared to the moderate to high differentiated EC group [29]. Especially in postmenopausal women, where there is no progestin antagonism of estrogen, the high level of 27HC may contribute to the development and progression of EC by activating ER receptors [30]. In addition, it was also found that 27HC may mediate the proliferation of EC cells by activating LXR, which provides a new approach to EC therapy by interfering with the target of LXR [29]. Furthermore, excessive aggregation of 27HC may lead to the activation of various inflammatory factors, such as IL-6 and tumor necrosis factor, which are involved in tumorigenesis and progression [31, 32].

#### LDL-C and EC

LDL-C is a group of cholesterol-rich lipoproteins that is mainly converted from VLDL and oxidatively modified to oxidized low-density lipoprotein (OX-LDL), which is a major contributor to atherosclerosis. Studies have shown that LDL is closely associated with various malignancies, such as breast, bladder, lymphomas, and rectal cancers, which may be related to increased LDL receptor (LDLR) activity and high LDL uptake in cancerous tissue cells [33–36]. The risk of EC is elevated by 1.3-fold when the serum LDL-C level is ≥ 100 mg/dL [25]. LDL, an essential nutrient, enables cancer cells to achieve rapid growth by mediating the uptake of energy from LDLR, accompanied by adipose tissue-induced generation of oxidative stress and thus leading to lipotoxicity, resulting in the production of large amounts of reactive oxygen species (ROS) [37, 38]. In turn, the oxidized LDL to OX-LDL in the presence of ROS can bind to CD36 and lectin-like oxidized LDL receptor (LOX-1) to further induce mutations, leading to the rapid growth of abnormal cells and the development and spread of cancer cells [39]. Moreover, ROS can also induce the release of various inflammatory factors that damage cellular DNA and alter the microenvironment for cell survival, thus contributing to tumorigenesis and mutation and inactivation of tumor suppressor genes [40]. In addition, ROS induce mutations in key tumor-associated genes such as RAS, EGFR, PGF, and HER2 [41]. In addition, high levels of ROS activate signaling pathways related to tumorigenesis, such as the PI3K and MAPK signaling pathways [42]. In recent studies, it was found that the use of LDL particles as anticancer drug carriers for the treatment of cancer not only reduces the side effects on humans but also enhances the toxic effects on cancer cells [43]. In summary, LDL-C is closely associated with EC pathogenesis, since the mechanism involved in EC is not fully understood, making antitumor drugs by interfering with LDL-C metabolism or exploiting the high uptake of LDL-C in tumor cells may be a new idea for the treatment of EC.

#### HDL-C and EC

HDL-C is a recognized “good density lipoprotein” and plays an important role in the cardiovascular system to fight against atherosclerosis caused by LDL-C. Current studies on HDL have suggested that HDL is strongly associated with the development and progression of several malignancies [44, 45]. It was found that the HDL level was significantly lower while the levels of TG and the ratio of TG/HDL were significantly higher in postmenopausal EC patients compared with normal controls, and after further correction of the data, the final results revealed that the larger the ratio, the higher the EC classification [8]. It is worth noting that there are two problems with this study; on the one hand, the experimental data were obtained from a review of medical records, and on the other hand, the experiment was conducted only on postmenopausal women, which has practical implications despite the limitations of the experimental design.

It was noted that higher levels of HDL in vivo played a pivotal role in reversing cholesterol transport (RCT), increasing the rate of cholesterol metabolism, and improving cellular metabolism in a highly lipotoxic environment [46, 47]. In addition, ApoA1, the main component of HDL-C, plays two important roles in the anti-inflammatory and antiproliferation of tumors. On the one hand, as an anti-inflammatory molecule, ApoA1 can directly interfere with T cells to inhibit monocyte activation and reduce the production of TNF-α and IL-1β, while on the other hand, it indirectly exerts antitumor proliferation by converting the M2 phenotype to the antitumor proliferative M1 phenotype in tumor-associated macrophages [48–50]. Although most studies have found that decreased HDL-C levels led to the development and progression of malignancies, there are also some studies pointing out that the link between HDL-C and malignancies is not clear, possibly due to the effects of multiple confounding factors such as obesity, diabetes, smoking, age, and alcoholism [47, 51]. In summary, the mechanism of the relationship between HDL-C and EC requires further study, which is extremely important for clinical applications and may serve as a means of detection and treatment of EC by targeting HDL-C metabolism (Table 2).

**Table 2:** Biological mechanisms between lipid metabolism and EC

Lipid Type	Mechanism or Pathway
HDL-C	HDL→ApoA I→T-cell→MNC→TNF-α, IL-1β↓→Inflammatory factors HDL→ApoA I→M2→M1↑→Antitumor
LDL-C	LDL→LDLR→ROS→PI3K/MAP↑→Carcinogenic effect LDL→LDLR→ROS→NF-κB→HER2 PGF RAS EGFR↑→Genetic mutations ROS→LDL-C→OX-LDL→CD36 LOX-1↑→Growth and metastasis
TC	TC→27HC→IL-6 TNF↑→Inflammatory factors TC→27HC→ER↑→Uterine epithelial cell proliferation TC→27HC→LXR↑→Growth and metastasis
TG	TG→SHBG + E→E↑→Angiogenesis and proliferation TG→PPARα↑→Carcinogenic effect TG→IHF→IR-A IGF-1R→PI3K/AKT ERK MAPK↑→Malignant phenotype

MNC, mononuclear cell; M1, macrophage 1; M2, macrophage 2; ROS, reactive oxygen species; LOX-1, lectin-like oxidized LDL receptor-1; 27HC, 27-hydroxycholesterol; LXR, liver X receptor; E, estrogen; ER, estrogen receptor; SHBG, sex hormone-binding globulin; IHF, impaired hepatic function; IR-A, insulin receptor-A; IGF-1R, insulin-like growth factor 1 receptor; “↑” represents positive regulation; “↓” represents negative regulation

### Biological Mechanisms and signaling pathways between lipid metabolism and EC

#### Obesity

Abnormal lipid metabolism is often accompanied by the presence of obesity, which is strongly associated with an increased risk of EC (RR of 1.52) [52]. It was noted that all-cause mortality increased by 9.2% per 10% increase in BMI, and it was 1.66 times higher in EC patients with BMI ≥ 40 than in those with BMI < 25 (95% CI: 1.10–2.51) [53]. Despite the presence of random variation, which leads to considerable heterogeneity among studies, it is important to emphasize that this heterogeneity is to be expected. As a result, these studies are still of great value. Adipose tissue is often considered an “endocrine organ”, and peripheral fat in obese patients is a source of estrogen in postmenopausal women, while in the absence of progestin antagonism, the endometrium will be chronically exposed to higher levels of estrogen, thereby promoting the occurrence and progression of EC. Meanwhile, high levels of estrogen may play a role in carcinogenesis by activating IGF-1 and EGRF, further activating the PI3K-AKT-mTOR signaling pathway to exert oncogenic effects [14, 54]. On the other hand, obese patients are often accompanied by IR and hyperinsulinemia, and higher insulin induces the expression of vascular endothelial growth factor receptor (VEGFR), which promotes angiogenesis as well as the proliferation of endometrial cells. In addition, adipokines secreted by adipose tissue, including leptin, monocyte chemotactic protein-1 (MCP-1), IL-6, IL-8, and TNF-α, can also promote tumorigenesis [55]. It is also noteworthy that the possibility of underdosing may exist in obese patients with EC, and underdosing may occur with the BSA calculation method of dosing compared with the method of weight-based dosing based on the Dubois formula [56–59]. Studies have found that there was a decrease in the risk of EC occurrence after bariatric surgery, with a summary OR of 0.21 (95% CI: 0.13–0.35) [60]. In conclusion, obesity is closely related to EC, and reasonable weight management may improve the morbidity and prognosis of EC. Research has demonstrated that the majority of adipokines exhibit pro-inflammatory properties, with the exclusion of adiponectin, which is a naturally occurring biopeptide that is secreted by adipocytes. The level of adiponectin, a hormone secreted by adipocytes, is observed to be decreased in obese patients, which may contribute to the heightened risk of endometrial cancer in this population. AdipoR1 and AdipoR2, the receptors for adiponectin, are widely distributed in uterine tissues. Upon binding to these receptors, adiponectin inhibits several signaling pathways, including JAK-STAT3, ERK1/2, and PI3K/AKT-mTOR, thereby exerting antiproliferative and proapoptotic effects [61].

#### Inflammation

Inflammation is closely linked to the occurrence and progression of cancer. In patients with abnormal lipid metabolism, it is usually accompanied by elevated levels of multiple adipogenic inflammatory factors, such as IL-1β, IL-6, IL-8, and TNFα [62]. Leptin plays an important role in the production of inflammatory factors in endometrial cells by elevating the expression of IL-1β, interleukin-1 receptor-associated kinase 1 (IRAK1), IL-6 and TNFα by regulating the PI3K/AKT3 and ERK1/2 signaling pathways [63, 64]. Studies have found that IRAK1 expression was increased in EC patients, mitosis in EC cells was inhibited after knockdown of IRAK1 [65], and phosphorylated IRAK1 might also activate NF-κB to mediate inflammatory responses. The elevated expression of IL-6 plays a role in mediating the ERK-NF-κB pathway to form an autocrine feedback loop to stimulate cell proliferation in EC cells [66]. Elevated TNFα expression can also significantly increase the risk of developing EC, with a 1.73-fold increase in EC risk (95% CI: 1.09–2.73) when the TNFα level > 1.328 pg/ml, which may be attributed to the effects on endometrial proliferation and angiogenesis by TNFα [67]. In addition, according to research findings, IL-8 can act on the G protein-coupled receptors CXCR1 and CXCR2, which are highly expressed on the EC cell membrane surface, and activate various signaling pathways, such as the MAPK and Akt signaling pathways, thereby promoting angiogenesis and cell proliferation, and these key inflammatory cytokines and pathways eventually contribute to EC development [68]. Of note, IL-6 and IL-11 have been shown in animal experiments to regulate the cell cycle as well as the migration of cells through the STAT3 pathway, and IL-11 is known to affect a variety of cellular targets, such as cytosolic protein 3, p21, Bcl-xL, Bcl-2, and survivin. STAT3 blockers reduce cell‒cell adhesion and disrupt the environment necessary for tumor development. Thus, further clinical studies of IL-6 and IL-11 may provide novel insights for the treatment of EC [69, 70].

#### Angiogenesis

Angiogenesis is one of the key mechanisms of the proliferation and invasion of cancer cells, which provide sufficient oxygen and nutrients for cancer growth and metastasis. The key role of hypoxia is to stimulate vascular production, and in hypoxia, hypoxia-inducible factor-1α (HIF-1α) is induced to become activated, leading to an increase in VEGF expression [71]. Angiogenesis is mainly induced by vascular endothelial growth factor (VEGF), which is secreted by various adipokines and inflammatory factors in adipose tissue [62]. In a study of obese mice, the results indicated that the VEGF-induced overactive mTOR signaling pathway was an important cause of EC progression. In addition, ps6 and AKT were found to be highly expressed in abnormal endometrium, which is considered a marker of mTOR activation, further confirming that the oncogenic effect exerted by high levels of VEGF is accomplished by stimulating the PI3K/AKT/mTOR pathway [72]. As a result, interference with the angiogenic effect of VEGF may serve as a valid target for EC therapy [73]. The drug bevacizumab has been approved for the treatment of a variety of malignancies in the United States by targeting angiogenesis in VEGF [74]. It is noteworthy that the findings from most of the current phase II clinical studies conducted with bevacizumab for EC indicated that treatment with carboplatin-paclitaxel combined with bevacizumab improved the prognosis of EC. Although the efficacy did not yet seem to be significant, the therapeutic effect exerted by bevacizumab is still of great research value and deserves further study in EC [75–77]. The angiopoietin-Tie receptor axis is an important angiogenic system in which angiopoietin-1 (Ang-2) and angiopoietin-2 (Ang-2) play important roles. It has been found that higher serum Ang-2 levels are associated with worse disease prognosis in patients with EC. Further studies showed that Ang-1 and Ang-2 expression was increased in tumor cells after VEGF-A/VEGFR2 signaling was blocked by bevacizumab. Combined silencing of both VEGF-A and Ang-2 will result in improved clinical outcomes for EC [71, 78, 79].

#### Estrogen metabolism

The normal menstrual cycle in women is divided into proliferative, secretory, and menstrual phases. During the proliferative phase, estrogen promotes the proliferation of endometrial cells, while in the secretory phase, the corpus luteum secretes progesterone, which exerts an antagonistic effect on estrogen and thus inhibits DNA synthesis and the cell cycle of endometrial cells. Before menopause, estrogen is mainly produced from ovarian cells, while in postmenopausal women with decreased ovarian function, estrogen is primarily converted from androgens by aromatase derived from adipose tissue metabolism in the body. However, since there is no progesterone that has an antagonistic effect on estrogen in postmenopausal women, the transformed estrogen will continue to stimulate endometrial cells and result in EC occurrence [61, 80]. At present, there are two sets of classification standards for EC. Molecular EC typing includes POLE hypermutant EC (POLEmut), defective EC mismatch (MMRd), EC p53 mutant (p53abn), and nonspecific EC molecular spectrum (NSMP), which classify EC based on gene mutations and immunohistochemistry [81, 82]. However, the impact of estrogen on these classifications is seldom addressed. In routine clinical management, a widely used classification criterion is histological grading, which categorizes EC into type I (estrogen-dependent) and type II (nonestrogen-dependent) grading [4]. There is a strong association between estrogen metabolism and type I EC, prompting further investigation into the etiologic relationship between estrogen and type I EC.

In addition, studies have shown that estrogen induces hepatocyte growth factor (HGF) expression in mesenchymal cells and further activates the c-Met-PI3K-Akt signaling pathway, ultimately leading to migration and invasiveness in EC cells. It is worth noting that TNFα can promote estrogen synthesis and enable E1 to convert into E2 with higher activity, resulting in a significant increase in HGF secretion in the above process [83, 84]. Highly active E2 plays a biological role through the classical receptors ERα and ERβ and G-protein-coupled ER and GPER. The biological functions of ERα and ERβ in endometrial tissues appear to be relative, with E2 promoting cell proliferation as well as invasion through activation of ERα and GPER, whereas ERβ inhibits this biological effect through degradation of ERα. This balance is disrupted in endometrial cancer tissues, where levels of ERα are threefold higher than ERβ. The exact biological effects of ERβ are unclear, but it is still worth noting [61, 85, 86]. Research has shown that ETS variant 4 (ETV4) exhibits significant expression in Ishikawa cells. Furthermore, approximately 50% of ETV4 specifically attaches to ER binding sites, playing a key role in the estrogen signaling pathway. Remarkably, when the ETV4 gene was knocked out, tumor growth was observed to decelerate by 1.6 times. This effect can possibly be attributed to the absence of ETV4 at ER binding sites, thereby impacting chromatin accessibility and estrogen receptor activation [87].

**Fig. 1:**
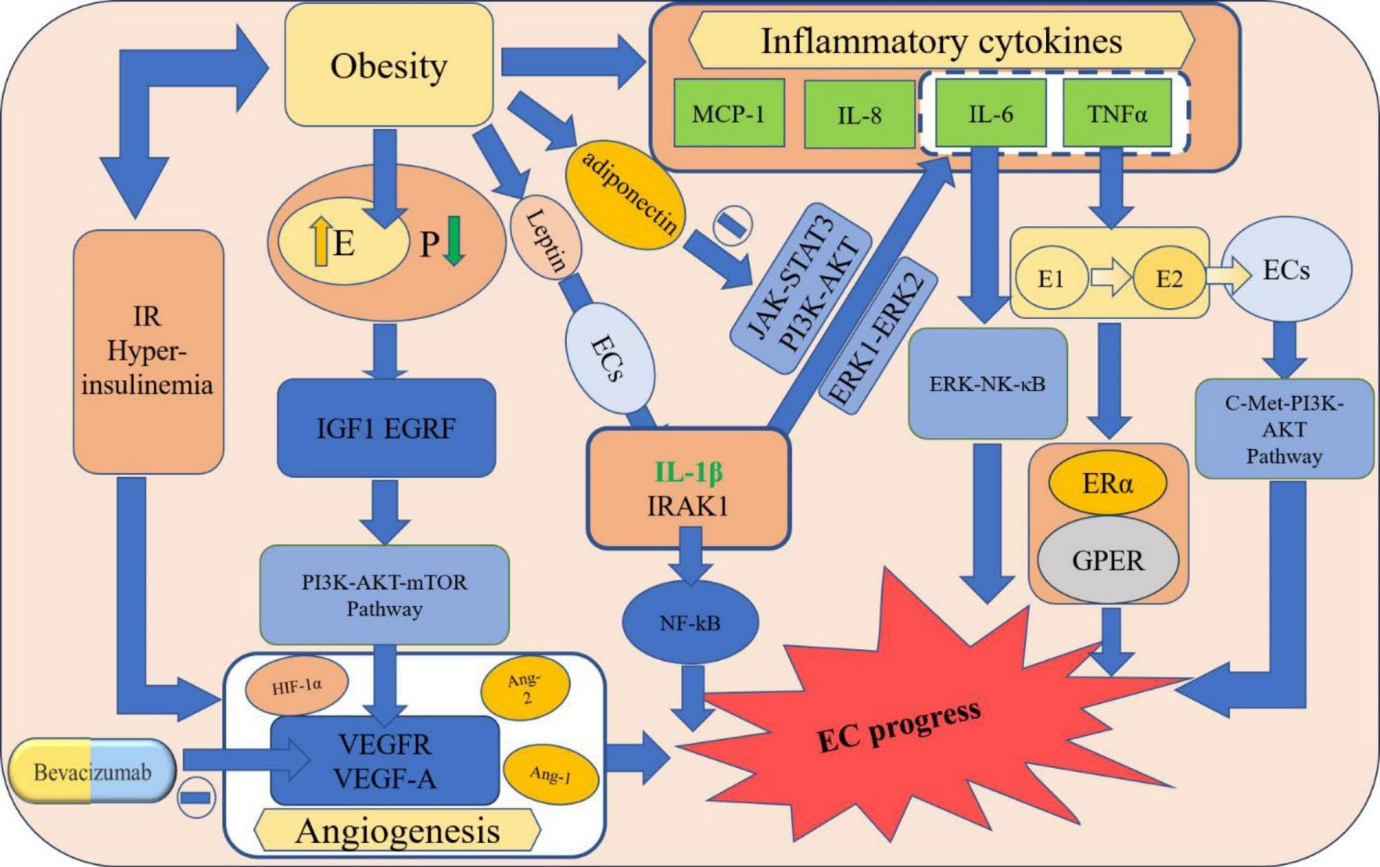
Biological mechanisms and signaling pathways between lipid metabolism and EC Yellow boxes indicate factors that are closely associated with the development of EC. Light blue boxes indicate signaling pathways. The green color indicates inflammatory factors. The yellow arrow indicates an increase. The green downward arrow indicates a decrease. IR: Insulin resistance; IGF1: Insulin-like growth factor 1; EGRF: epidermal growth factor receptor; ECS: endometrial cells; VEGFR: vascular endothelial growth factor receptor; E: estrogen; E1: estrone; E2: estradiol; P: progesterone. IRAK1: interleukin-1 receptor-associated kinase 1. HIF-1α: hypoxia-inducible factor-1α; Ang: angiopoietin; GPER: G protein-coupled estrogen receptor. The dotted box indicates the pathways through which each inflammatory factor acts

Taken together, estrogen metabolism, obesity, inflammatory factors, and the angiogenesis process interact with each other to promote the development and progression of EC(Fig. 1). Therefore, studying their interactions may provide effective therapeutic strategies for EC.

## Potential therapeutic mechanisms of statins in EC

Recent studies have confirmed the close relationship between dyslipidemia and EC, and scientists are increasingly concerned about the role of lipid-lowering drugs in EC. The types of lipid-lowering drugs currently used in clinics include statins, fibrates, nicotinic acid, ezetimibe, and bile acid chelators, with statins being the most commonly used. Studies on the application of statins have found that long-term use of statins reduced the incidence of EC, with a summary OR of 0.59 (95% CI: 0.40–0.87). Furthermore, its use in newly diagnosed EC patients significantly decreased the mortality rate, with a summary HR of 0.43 (95% CI: 0.29–0.65) [88, 89].

**Fig. 2:**
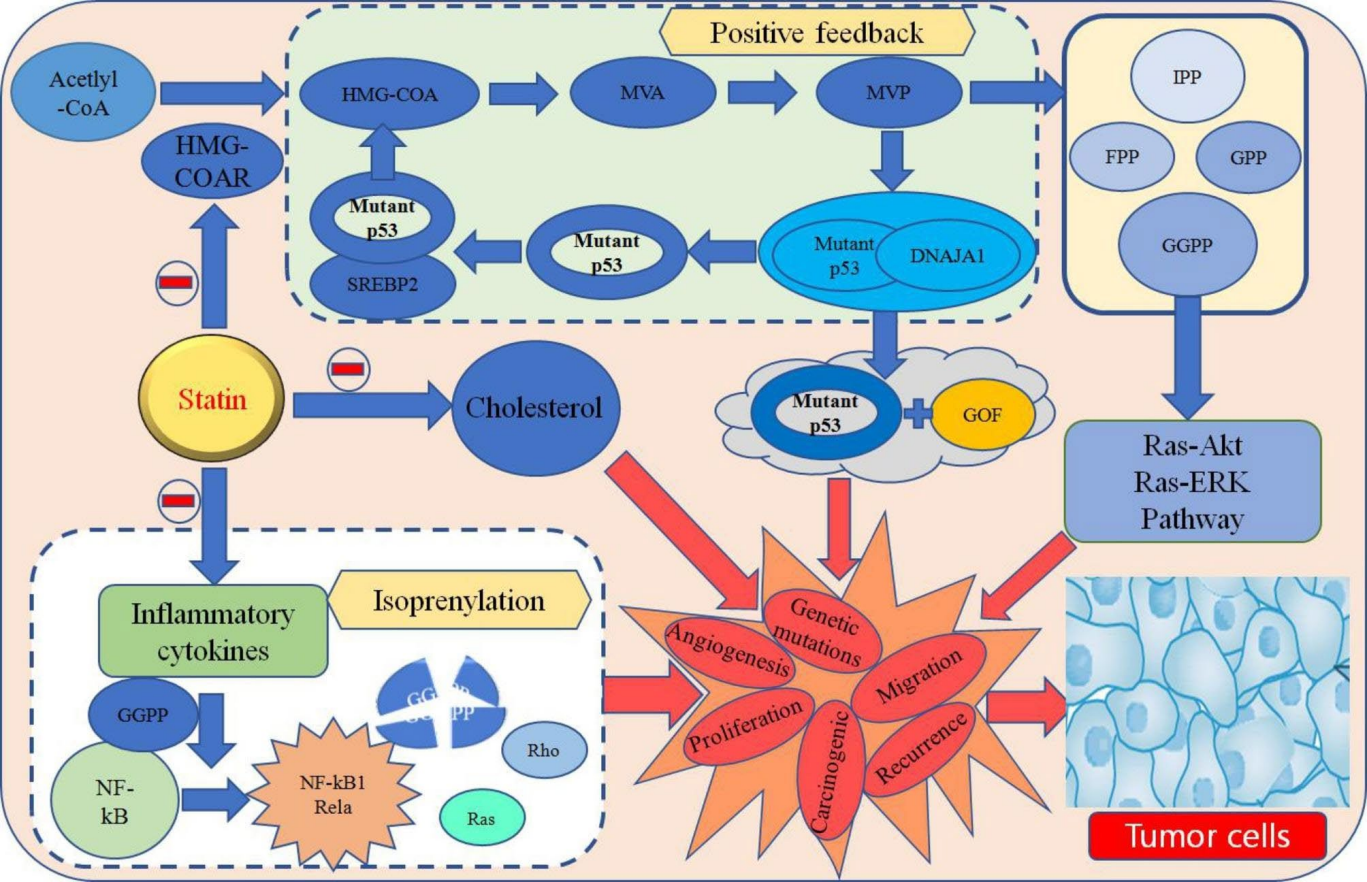
Potential therapeutic mechanisms of statins in EC “-” represents negative regulation; the red arrow indicates the effect on cancerous tissue. DNAJA1: DnaJ heat shock protein family (Hsp40) member A1; GOF: gain of function; SREBP2: Sterol-regulatory element binding protein 2

Statins lead to a reduction in cholesterol production and exert lipid-lowering effects primarily by inhibiting HMG-COA reductase (HMGCR), a key enzyme in cholesterol synthesis. Studies have indicated that the mevalonate pathway involved in cholesterol synthesis is closely related to cancer development by producing a variety of raw materials necessary for protein modification, and an increased mevalonate kinase requirement has become a hallmark of cancer progression [90]. Moreover, HMGCR is highly expressed in EC cells, and statins may exert anticancer effects by inhibiting HMGCR expression and the mevalonate pathway [91]. The mevalonate pathway begins with acetyl coenzyme A, and the metabolic intermediates include HMH-COA, mevalonate (MVA), mevalonate-5-phosphate (MVP), isopentenyl pyrophosphate (IPP), geranyl pyrophosphate (GPP), farnesyl pyrophosphate (FPP), geranylgeranyl pyrophosphate (GGPP), and cholesterol. Mutant p53 is the most common mutant genotype in human malignancies and can exhibit oncogenic activity through gain of function (GOF) [92]. MVP can promote the interaction of mutant p53 with DNAJA1 (DnaJ heat shock protein family (Hsp40) member A1) to maintain the stability of mutant p53, and mutant p53 will, in turn, bind SREBP2 (sterol-regulatory element binding protein 2) to promote the mevalonate pathway, further increasing MVP expression to form positive feedback and thus making mutant p53 more stable [93]. Second, FPP and GGPP produced in the mevalonate pathway can participate in the isoprenoidation process of hundreds of proteins, and the inhibition of HMGCR causes a decrease in the concentration of FPP and GGPP and interferes with the isoprenylation of the Ras and Rho families, which is a key mechanism that may be important for statins in the treatment of cancer [94, 95]. In addition, low levels of GGPP also inhibit the Ras/Akt and Ras/ERK signaling pathways and affect the expression of VEGF [96]. Furthermore, statins have an effective anti-inflammatory function by inhibiting the transcriptional activity of NF-κB, and both active forms of NF-κB, NK-κB1 (p50) and RelA (p65), require the involvement of GGPP in isoprenylation to fully exert their effects [97] (Fig. 2). Most importantly, statins can also directly inhibit cholesterol production and disrupt the highly nutritious environment that rapid tumor growth requires. A large body of research has now supported the efficacy of statins for the treatment of EC, but many questions remain to be answered [98–101]. Larger doses of statins may be required to produce biological activity, which inevitably raises a question about their use in oncologic treatment. Statins, which may cause transverse myolysis, are more likely to be toxic to muscle at higher doses, and while the incidence is less than 1 in 10,000, it is still very severe and is an issue that current research needs to address. For this reason, studies of statins in EC therapy should better discuss drug toxicity as well as issues of tolerability and clarify the timing of statin use as well as medication combination [102].

Taken together, these potential therapeutic mechanisms of statins may provide new insights for clinicians in preventing EC and treating dyslipidemia combined with EC. However, multicenter clinical studies with large sample sizes are still needed to further provide evidence for more effective application in clinical practice.

## Strengths and limitations

Here, we provide a comprehensive review of recent studies of the etiological relationship between dyslipidemia and EC in terms of lipid metabolism and associated biological mechanisms associated with lipid metabolism, with a significant body of literature and good data relativity. In addition, the paper discusses the possibility of statins as adjuvant therapy for EC, providing new ideas for EC treatment. There is a large amount of epidemiological evidence in the review, but the epidemiological evidence is prone to confounding factors and is influenced by a variety of conditions. Therefore, the study of lipid metabolism and EC pathogenesis should be more in depth, and it is particularly important to study pathogenesis in clinical trials to provide increasingly definitive evidence on lipid metabolism and EC pathogenesis. The review discussed the etiological relationship between dyslipidemia and EC in terms of EC histological classification but not from the perspective of molecular typing, and the deeper etiological relationship between dyslipidemia and molecular typing has not been clarified. Therefore, a more definite mechanism of action remains to be explored in greater depth, which is where attention should be given.

## Conclusions

In this review, we focus on the correlation between lipid metabolism and EC and discuss the evidence that abnormal lipid metabolism promotes an increase in EC growth and metabolism, as well as the regulatory mechanism and related signaling pathways involved in this relationship. Our findings highlight the significant role played by various factors, such as low levels of HDL, high levels of TC, LDL, TGs, and obesity, in both the development and progression of EC. These factors contribute to a poorer prognosis for EC patients. Therefore, it is clinically possible to identify EC patients at an early stage by measuring serum lipid levels. This early detection enables patients to receive early treatment, reducing the risk of tumor metastasis and ultimately improving the prognosis of EC. In clinical practice, the initial prognosis of EC patients can also be assessed by evaluating serum lipid levels. The more severe the disorder of lipid metabolism, the poorer the prognosis for EC. Furthermore, we explore the potential benefits of statins that act on lipid metabolism as an adjunctive therapy for EC. Statins have shown promise in improving patient prognosis and reducing mortality rates. However, extensive clinical trials and mechanistic studies are needed to establish the true effectiveness of statins in the treatment of EC.

This review offers a comprehensive perspective to enhance a better understanding of the etiological relationship between lipid metabolism and EC and further open up new therapeutic possibilities and effective treatments for EC by targeting lipid metabolism.

## Data Availability

Data sharing does not apply to this article, as no datasets were generated or analyzed during the current study.

## Abbreviations

EC: Endometrial carcinoma
TG: Triglycerides
TC: Total cholesterol
LDL-C: Low-density lipoprotein cholesterol
HDL-C: High-density lipoprotein cholesterol
IR: Insulin resistance
